# ASSESSMENT OF AGE INCLUSIVITY: RESULTS FROM A RESEARCH-INTENSIVE COLLEGE OF NURSING

**DOI:** 10.1093/geroni/igae098.1590

**Published:** 2024-12-31

**Authors:** Jacqueline Eaton, Katarina Friberg-Felsted, Summer Martin, Rebekah Perkins, Kara Dassel, Sara Hart

**Affiliations:** University of Utah, Salt Lake City, Utah, United States; University of Utah, Salt Lake City, Utah, United States; University of Utah, Salt Lake City, Utah, United States; University of Utah, Salt Lake City, Utah, United States; University of Utah, Salt Lake Cty, Utah, United States; University of Utah, Salt Lake City, Utah, United States

## Abstract

Age inclusivity is crucial for meeting the needs of an aging population. However, in higher education, age-stratification reinforces categorization of traditional and nontraditional students. This reduces the ability to educate and prepare trainees for work with older adults upon graduation. Seeking to improve educational opportunities at the University of Utah College of Nursing, a needs assessment was conducted using the Age-Friendly Inventory (AFI) and Campus Climate Survey (CCS). The outcomes of the CCS assessment are reported here. All faculty, staff, and students in the College of Nursing were invited to access the CCS e-survey via email or QR code. The survey was available during the months of October and November 2023. During this time, the research team used e-flyers, presentations at college meetings, and invitations from administration to increase response rate. Data was managed using Qualtrics and analyzed using descriptive statistics to identify perceptions of age-friendliness and beliefs about age inclusivity. A total of 247 individuals completed the survey. Students made up the majority of participants (63.6%); nineteen percent were faculty and 17% were staff. The majority (53%) were unfamiliar with the age-friendly university initiative. Ageism was seen as a major problem in society by 80.8% of the sample, yet only 29.4% believe it to be a problem on campus. Overall, 14.7% percent perceived our campus as age-friendly, which implies a need to increase knowledge about age inclusivity and age-friendly practices. Strategic planning is underway to outline specific initiatives to improve perceptions and practices within the College of Nursing.

